# A cross-sectional survey of work and income loss consideration among patients with herpes zoster when completing a quality of life questionnaire

**DOI:** 10.1186/s12913-018-3451-9

**Published:** 2018-08-25

**Authors:** Kelly D. Johnson, Susan K. Brenneman, Chrisann Newransky, Seth Sheffler-Collins, Laura K. Becker, Angela Belland, Camilo J. Acosta

**Affiliations:** 1 Center for Observational and Real World Evidence (CORE), Merck & Co., Inc, 351 North Sumneytown Pike, UG2AB-30, North Wales, PA 19454 USA; 2 Health Economics and Outcomes Research, Optum, 11000 Optum Circle, Eden Prairie, MN 55344 USA; 3 Merck Research Laboratories, 351 North Sumneytown Pike, North Wales, PA 19454 USA

**Keywords:** Herpes zoster, Quality of life, Work loss, Income loss, Productivity

## Abstract

**Background:**

Prior research suggests that many patients do not spontaneously include work/income loss when responding to utility assessments, although this remains unconfirmed in the US due to almost no published US-based studies to date, and has not been previously studied among patients with herpes zoster (HZ). The objective of this study was to examine whether patients with HZ consider work and income loss when completing a quality of life survey.

**Methods:**

A cross-sectional survey was administered to 2000 US adult commercial health plan enrollees aged 50–64 years with ≥ 1 HZ medical claim during 2014. The survey collected information related to health status (EQ-5D), work productivity, and HZ severity and clinical features.

**Results:**

Mean respondent age was 58.4 years [standard deviation (SD) 4.1] and 62.0% were female. About 3 in 4 (76.8%) patients (*N* = 772) were employed either full (69.9%) or part time (6.9%). Less than half (45%) spontaneously considered work/income loss when responding to EQ-5D, and mean EQ-5D scores for patients who considered work/income loss were lower than for patients who did not [0.56 (SD = 0.28) vs. 0.69 (SD = 0.24); *p* < 0.001]. Overall, 43% of patients reported at least one full day missed (mean = 9 full days) and 29% reported at least one partial day missed (mean = 6 partial days) during the most recent shingles episode. Patients who considered work loss were more likely to have missed full (76.4% vs 26.0%, *p* < 0.001) or partial (70.9% vs. 35.2%, *p* < 0.001) days. Patients with absenteeism were more likely to consider work/income loss when completing EQ-5D [odds ratio (OR) = 7.91, 95% confidence interval (CI) 5.01–12.31]. Odds of absenteeism/presenteeism increased significantly with increasing levels of HZ severity, and higher odds were associated with pain located on the face/scalp/neck/eye/ear (OR 1.90, 95% CI 1.06–3.40) and with pain lasting 12+ months (OR = 2.91, 95% CI 1.14–7.42).

**Conclusions:**

HZ has considerable impact on the work and productivity of adults aged 50–64 years old. However, many patients with HZ do not spontaneously consider work/income loss when completing a standardized quality of life questionnaire. Studies that use health state utilities in HZ based on EQ-5D may not fully reflect the societal costs of work loss.

**Electronic supplementary material:**

The online version of this article (10.1186/s12913-018-3451-9) contains supplementary material, which is available to authorized users.

## Background

Herpes Zoster (HZ) (“shingles”) results from the reactivation of varicella-zoster virus located in sensory ganglia. Usually HZ is acute and patients experience mild to moderate pain that can have substantial physical and psychological impact. About 10–15% of patients with HZ subsequently develop post-herpetic neuralgia (PHN), a persistent, chronic neuropathic pain [1]. The estimated overall annual cost of HZ in US (2013) among persons aged 50 or older has been estimated at $1.85 billion (B) in direct costs and an additional $3.2B in indirect costs, including productivity loss [2]. Work loss (“absenteeism”) per HZ episode is reported by 50–64% of patients, with 51–76% reporting reduced productivity at work (“presenteeism”) [3, 4]. HZ has a negative impact on patient quality of life, regardless of patient age [5–8].

An HZ vaccine (Zostavax®) was licensed by the US FDA in 2006 and is recommended for routine use among adults aged 60 and older by the US Centers for Disease Control and Prevention (CDC) Advisory Committee on Immunization Practices (ACIP) [9, 10]. Zostavax® was also approved for use among adults aged 50–59 years in 2011, but routine use is not currently recommended by the ACIP. Although several studies have demonstrated a sharply continuing rise in incidence of HZ beyond age 50 [11–13] evidence is lacking regarding cost-effectiveness of HZ vaccination among persons aged 50–59 years. HZ episodes are generally less severe for younger age groups, so the benefits of HZ vaccination may be more difficult to demonstrate. Even so, patients who are 50–59 years of age may be more likely to have HZ impact their work and productivity than older patients; 72.1% of persons aged 50–59 years were employed in the US in 2015, compared to 28.4% of those aged 60 years or older [14].

Research suggests that many patients do not spontaneously include work/income loss when responding to utility assessments [15]. Therefore, health-state utility evaluation, including those based on standardized scales such as the EuroQol Five Dimensions Questionnaire (EQ-5D) [16, 17], may not fully reflect the impact of work loss. Consequently, it may be appropriate to account for this effect separately in the cost calculations [18]. A review of the literature regarding work/income loss consideration in health-state utility assessments across health conditions indicates mixed findings [19–28]. The most recent systematic review on this topic was published in 2010, and it concluded that more empirical work was required on the topic using generic quality of life instruments and larger samples [15]. Furthermore, almost all assessments of the relationship between work/income loss consideration and health-state utilities have been conducted outside the US. Since public/private social safety nets (e.g. paid sick leave, unemployment, etc.) may differ greatly by country, assessing country-specific utility values are crucial [29]. Overall, whether consideration of lost income is included in health state valuations (and subsequent economic evaluations) in the US remains largely unknown.

The primary objective of this study was to examine whether patients who have experienced HZ consider income and productivity loss when completing a generic quality of life survey. Secondary objectives included the following: 1. to assess the impact of HZ on absenteeism or presenteeism among working adults; and 2. to assess the associations among EQ-5D scores, the severity of HZ, whether a patient experiences absenteeism or presenteeism, and whether the patient considers income and productivity loss when completing EQ-5D.

## Methods

### Study design

This study was a cross-sectional survey which utilized the Optum research database of medical and pharmacy claims linked to enrollment information to identify patients with HZ. Following identification, eligible patients were invited by mail to participate in the survey.

### Sample size

To estimate the sample size of subjects required for the survey portion of the study, the primary outcome measure was assumed to be the proportion of survey respondents that indicated they considered work and/or income loss when completing the first presentation of the EQ-5D. A target sample size was determined by the value and desired precision of measured proportions for this outcome variable. Because the actual proportions were unknown, the largest sample size required for a precision of ±0.05 for all values of proportions was targeted (*n* = 385). Assuming a 20% response rate a priori, 2000 patients were contacted to participate in the survey to obtain an evaluable sample of at least 400 completed patient surveys.

### Study population

Patients aged 50–64 years were identified via at least 1 claim with an International Classification of Diseases, Ninth Revision, Clinical Modification (ICD-9-CM) code for HZ (053.0–053.9) in any position between January 1, 2014 and December 31, 2014. The first qualifying HZ claim was identified as the index claim, and the date of that first claim was the index date. Patients were required to have continuous health plan enrollment 9 months prior to and 3 months subsequent to the index date. From this preliminary patient population, 2000 patients were selected to receive the study survey as follows. To ensure a study sample of patients that included more severe HZ, all eligible patients with evidence of nervous system complications (ICD-9-CM 053.1x) and/or eye complications (ICD-9-CM 053.2x, 053.71) were automatically included in the final sample of 2000 and were categorized as “more severe” patients. Patients from the remaining preliminary population (ICD-9-CM 053.0, 053.8, 053.79, 053.9) were categorized as “less severe” and were randomly selected to complete the final surveyed sample of 2000 patients. Identified patients were invited by mail to participate, and a process for mailed surveys developed by Dillman et al. was followed [30].

### Survey-based data

The data collection instrument included questions related to demographic and HZ clinical characteristics, health status (EQ-5D questions regarding dimensions of mobility, self-care, usual activities, pain/discomfort, anxiety/depression, with answers describing no, moderate, or extreme impairment; and overall health state scale) [16, 17], and work experience during the most recent HZ episode. Patient demographic characteristics included marital status (married/living with partner, widowed, divorced, separated, never married), highest level of education, employment status, race, and income. HZ clinical characteristics included severity (very mild, mild, moderate, severe, or very severe), worst level of pain experienced (11-point scale; 0 = no pain and 10 = worst possible pain), duration of pain (scale ranging from < 1 week to ≥ 12 months) and location of HZ outbreak (obtained via indication on a body figure drawing). The EQ-5D [16, 17] was presented with instructions that advised the patient to recall a day during the most recent shingles episode and answer health state questions as he/she would have on that day. Survey questions regarding work experience included number of full or partial days missed (absenteeism), rating of effectiveness (presenteeism) at work (0% “not effective at all” to 100% “completely effective”), reasons for absence, and use of paid sick, vacation or disability days.

### Primary and secondary outcome measures

The primary outcome variable was calculated as the proportion of patients who answered that they considered work/income loss when responding to EQ-5D questions. Secondary outcomes included the EQ-5D index scores, absenteeism and presenteeism. EQ-5D health states were converted into a single summary index by applying weights to each of the levels (1 = no problems, 2 = some problems, 3 = extreme problems) in each dimension according to the standard instructions provided by the EuroQoL Group [31]. An EQ-5D summary index was calculated for each subject. For each EQ-5D dimension, dichotomous variables were created indicating whether it was at least a moderate problem or a severe problem. These variables were combined to create 16 utility factors, which were multiplied by a US-specific preference weight, aggregated, and subtracted from 1 to form utility scores. If any dimension was missing, the utility score was assigned a missing value. The tariff used to transfer the EQ-5D to utility scores was based on Shaw et al. representing a US population using the time trade-off (TTO) technique [32]. The proportion of patients who experienced absenteeism or presenteeism was calculated. Absenteeism was defined as patient self-reporting of at least 1 entire or 1 partial day of work loss due to shingles, while presenteeism was defined as self-reported effectiveness at work of ≤ 70%. No work loss was defined as patient self-reported effectiveness at work as 80–100% and no reported full or partial lost work days.

### Claims-based data captured

Following completion of survey data collection, pharmacy and medical claims data for the 9 months preceding and 3 months following the index date were extracted and merged to the survey data. Baseline demographic characteristics included patient age (calculated as of index year), gender, and geographic region. Baseline clinical characteristics (during a baseline period of 9 months period preceding the index date) included the Quan-Charlson comorbidity score [33], which was calculated for each patient to measure overall comorbidity burden, and comorbid chronic conditions, identified using ICD-9-CM codes and the Clinical Classification software managed by AHRQ [34]. Indicator variables corresponding to specific HZ ICD-9-CM codes were created using medical claims during the study identification period in order to capture HZ complications. HZ treatment with antiviral medication during the 3-month period following the index date was also assessed.

### Statistical analysis

The analytic population consisted of all survey respondents with completed surveys, including all EQ-5D items. Descriptive statistics were calculated for all measures, including means, medians, and standard deviations (SD) for continuous variables and frequency distributions for categorical variables. Bivariate comparisons of patient characteristics included t-tests for continuous measures and chi-squared tests for categorical measures. Multivariate analysis was conducted using regression models appropriate for each dependent variable assessed based on the distribution of the measure. For each model, specific predictors to be included were determined based upon clinical rationale and statistical significance. Consideration of work/income loss (yes/no) as the dependent variable was modeled using logistic regression to assess the impact of actual absenteeism or presenteeism, adjusted for HZ severity, pain location and duration; employment status; age; and gender. Absenteeism/presenteeism vs. no work loss as the dependent variable was also modeled using logistic regression, and covariates included HZ severity, pain location and duration, employment status, age, and gender.

### Regulatory considerations

All data were used in compliance with state and federal regulations related to the privacy and security of individually identifiable health information, including the Health Insurance Portability and Accountability Act (HIPAA) Standards for Privacy of Individually Identifiable Health Information. Independent study approval was obtained from the New England Institutional Review Board and a waiver of documentation of informed consent was granted. The mailed study packet informed patients that their consent for participation was implied by returning the completed survey.

## Results

**Fig. 1:**
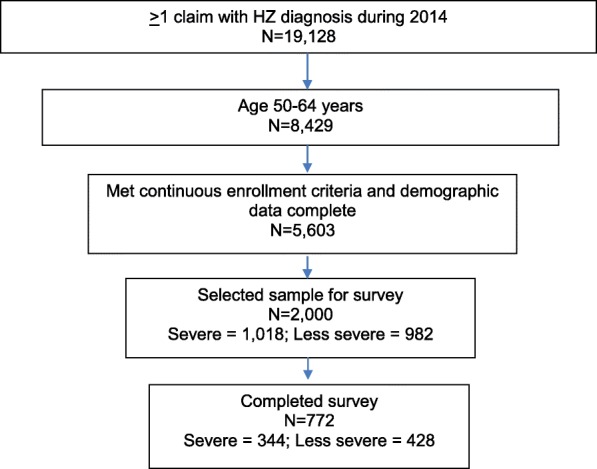
Survey sample and cohort attrition. Includes a flowchart diagram illustrating the study population identification and survey sample selection process. The flowchart begins with the total number of preliminarily eligible patients with ≥ 1 administrative claim with an HZ diagnosis during 2014 (*N* = 19,128), and depicts the number of patients selected at each step of the cohort identification and survey administration process to result in a final study sample of 772 patients

Among 19,128 patients with at least one HZ claim during the calendar year 2014, 5603 met all study entry criteria (Fig. 1). From this preliminary population, 2000 patients (1018 severe and 982 less severe) were selected as described above to receive the study survey. A total of 772 patients completed the survey, corresponding to an overall response rate of 43%. This response rate was calculated using methods described by the American Association for Public Opinion Research [35].

Baseline patient characteristics are included in Table 1. Mean age overall was 58.4 years (SD 4.1) and 62.0% were female. Most patients (90.9%) were white and the majority were married with at least a high school education. About 3 in 4 (76.8%) were employed either full- (69.9%) or part-time (6.9%). Within 3 months of the index date, 71.4% of respondents had evidence of HZ antiviral therapy. The most commonly used agent was valacyclovir. Overall, 34.6% of patients rated their HZ as severe or extremely severe (Table 2); 32.0% of patients had HZ with nervous system complications, and 23.1% had HZ with eye complications, based on ICD-9-CM diagnosis codes from claims data (Table 1).

**Table 1:** Baseline patient demographic and clinical characteristics

Characteristic	
*N*	772
Age, mean (SD)	58.4 (4.1)
Female, *n* (%)	478 (62.0)
Race/ethnicity, *n* (%)	
American Indian/Native Alaskan	7 (0.9)
Asian/Pacific Islander	18 (2.3)
Black or African American	30 (3.9)
White	700 (90.9)
Other	21 (2.7)
Hispanic/Latino	35 (4.6)
Married/Living with Partner, *n* (%)	606 (78.5)
Employment status, *n* (%)	
Full-time employment	536 (69.9)
Part-time employment	53 (6.9)
Not employed/working for pay	28 (3.7)
Retired	98 (12.8)
Homemaker	52 (6.8)
Income, *n* (%)	
Less than $25,000	36 (4.9)
$25,000 - $49,999	122 (16.7)
$50,000 - $74,999	167 (22.8)
$75,000 - $99,999	115 (15.7)
More than $100,000	292 (39.9)
Any claims-based evidence of HZ treatment	551 (71.4)
Acyclovir	156 (20.2)
Famciclovir	44 (5.7)
Valacyclvir	370 (47.9)
Identification diagnosis code (ICD-9-CM), *n* (%)	
053.0; Herpes zoster with meningitis	4 (0.5)
053.1×; Herpes zoster with nervous system complications	247 (32.0)
053.2×; Herpes zoster with eye complications	178 (23.1)
053.7×; Herpes zoster with ear complications	14 (1.8)
053.71; Otitis externa due to herpes zoster	2 (0.3)
053.79; Other specified herpes zoster complications	12 (1.6)
053.8; Unspecified herpes zoster complication	9 (1.2)
053.9; Herpes zoster without mention of complication	572 (74.1)

**Table 2:** Patient-reported herpes zoster characteristics stratified by consideration of work/income loss

	Total^a^ (*N* = 772, 100%)			Considered = YES (*N* = 347, 44.9%)			Considered = NO (*N* = 425, 55.1%)			*p*-value
Herpes Zoster Severity	valid *N*	*n*	%	valid *N*	*n*	%	valid *N*	*n*	%	
Very mild	769	108	14.04	345	31	8.99	424	77	18.16	< 0.001
Mild	769	156	20.29	345	47	13.62	424	109	25.71	< 0.001
Moderate	769	239	31.08	345	115	33.33	424	124	29.25	0.223
Severe	769	192	24.97	345	105	30.43	424	87	20.52	0.002
Extremely severe	769	74	9.62	345	47	13.62	424	27	6.37	< 0.001
Length of time pain lasted categories	valid *N*	*n*	%	valid *N*	*n*	%	valid *N*	*n*	%	
Less than 1 month	770	383	49.74	347	154	44.38	423	229	54.14	0.007
1 to less than 3 months	770	215	27.92	347	101	29.11	423	114	26.95	0.507
3 months to less than 6 months	770	61	7.92	347	29	8.36	423	32	7.57	0.685
6 months to less than 12 months	770	42	5.45	347	21	6.05	423	21	4.96	0.509
12+ months	770	69	8.96	347	42	12.10	423	27	6.38	0.006
Location of pain^b^										
Face/Scalp/Neck/Eye/Ear	771	326	42.28	346	161	46.53	425	165	38.82	0.031
Shoulder/Upper Arm/Elbow/Forearm/Hand	771	106	13.75	346	43	12.43	425	63	14.82	0.337
Chest/Abdomen/Upper Back/Lower Back	771	387	50.19	346	169	48.84	425	218	51.29	0.499
Groin area/Buttocks	771	71	9.21	346	40	11.56	425	31	7.29	0.042
Thigh/Knee/Shin/Calf/Ankle/Foot	771	76	9.86	346	34	9.83	425	42	9.88	0.979
	*n*	mean	SD	*n*	mean	SD	*n*	mean	SD	
Average level of pain	772	5.60	2.57	347	6.23	2.60	425	5.08	2.43	< 0.001
Worst level of pain	769	7.15	2.71	346	7.79	2.50	423	6.61	2.76	< 0.001

^a^Categories based on responses to Q8. “Did you consider work/productivity loss when you answered the health questionnaire?”

^b^Subjects can report more than one location

### Primary outcome: Work/income loss consideration

**Fig. 2:**
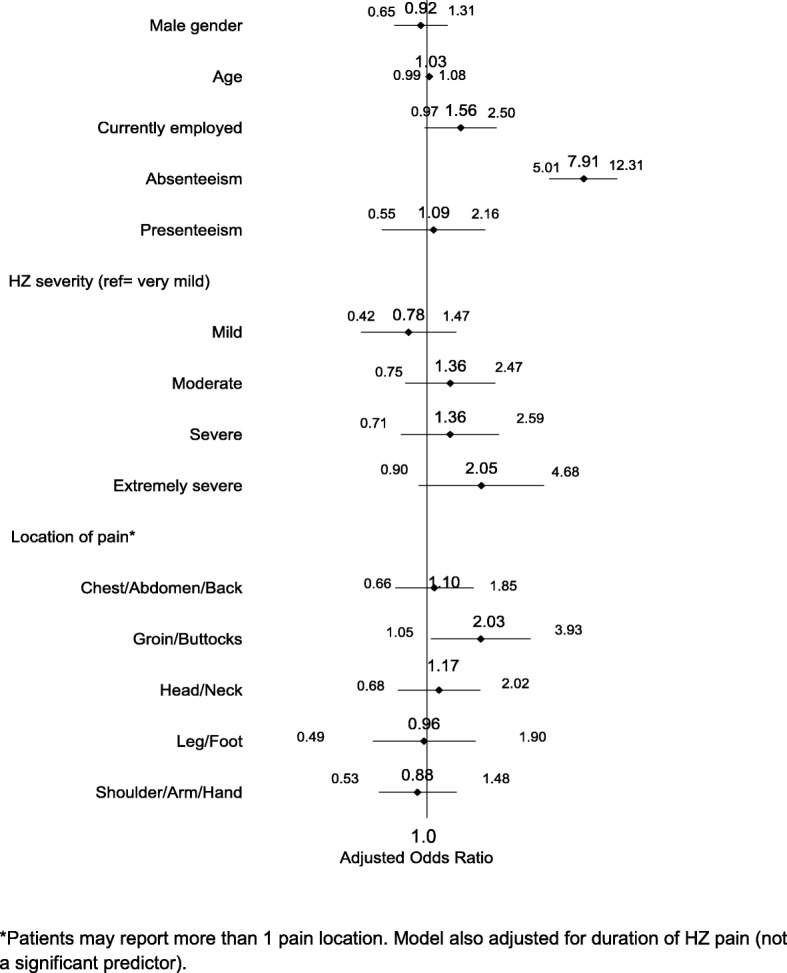
Odds of work/income loss consideration when completing EQ-5D. Illustrates the adjusted odds ratios of work/income loss consideration when completing the EQ-5D. Patients with absenteeism during the HZ episode were more likely to consider work/income loss when completing the EQ-5D (OR = 7.91, 95% CI 5.01–12.31), while presenteeism was not significantly associated with consideration of work/income loss (OR = 1.09, 95% CI 0.55–2.16)

Fewer than half (44.9%) of survey respondents indicated that they considered work/income loss when answering EQ-5D questions (Table 2). Patients who considered work/income loss reported greater severity of HZ, were more likely to have pain lasting > 12 months and less likely to have pain lasting < 1 month, have HZ located on face/scalp/neck/head or groin/buttocks, and have higher mean pain levels. Mean EQ-5D scores for those who reported that they did consider work/income loss were significantly lower than for patients who reported they did not consider [0.56 (SD 0.28) vs 0.69 (SD 0.24), *p* < 0.001] (Table 3). Significant differences were observed across all domains, but the largest impacts were observed for the ‘usual activities’ domain (level 3 = 27.2% vs 7.8%, *p* < 0.001) and the ‘pain/discomfort’ domain (level 3 = 43.6% vs 24.4%, *p* < 0.001). Patients with absenteeism during the HZ episode were more likely to consider work/income loss when completing the EQ-5D [odds ratio (OR) =7.91, 95% confidence interval (CI) 5.01–12.31] (Fig. 2). Presenteeism during the HZ episode was not significantly associated with consideration of work/income loss (OR = 1.09, 95% CI 0.55–2.16).

**Table 3:** EQ-5D scores* stratified by work loss consideration and absenteeism/presenteeism

	Total (*n* = 772)	Considered Work Loss (*n* = 347)	Did Not Consider Work Loss (*n* = 425)	*p* value	Absenteeism (*n* = 433)	Presenteeism only (*n* = 78)	No work loss (*n* = 222)	*p*-value
Index Score, mean (SD)	0.63 (0.27)	0.56 (0.28)	0.69 (0.24)	< 0.001	0.56 (0.27)	0.62 (0.25)	0.79 (0.18)	< 0.001
Mobility, *n* (%)								
Level 1	551 (71.7)	219 (63.5)	332 (78.5)	< 0.001	281 (65.4)	53 (68.0)	193 (87.3)	< 0.001
Level 2	164 (21.4)	93 (27.0)	71 (16.8)	< 0.001	107 (24.9)	22 (28.2)	25 (11.3)	< 0.001
Level 3	53 (6.9)	33 (9.6)	20 (4.7)	0.009	42 (9.8)	3 (3.9)	3 (1.4)	< 0.001
Self-Care, *n* (%)								
Level 1	596 (77.5)	244 (70.5)	352 (83.2)	< 0.001	310 (71.9)	57 (73.1)	202 (91.4)	< 0.001
Level 2	158 (20.6)	94 (27.2)	64 (15.1)	< 0.001	111 (25.8)	20 (25.6)	18 (8.1)	< 0.001
Level 3	15 (2.0)	8 (2.3)	7 (1.7)	0.512	10 (2.3)	1 (1.3)	1 (0.5)	0.200
Usual activities, *n* (%)								
Level 1	316 (41.1)	83 (24.0)	233 (55.1)	< 0.001	113 (26.2)	27 (34.6)	162 (73.3)	< 0.001
Level 2	326 (42.4)	169 (48.8)	157 (37.1)	0.001	211 (49.0)	43 (55.1)	55 (24.9)	< 0.001
Level 3	127 (16.5)	94 (27.2)	33 (7.8)	< 0.001	107 (24.8)	8 (10.3)	4 (1.8)	< 0.001
Pain/Discomfort, *n* (%)								
Level 1	64 (8.3)	24 (7.0)	40 (9.5)	0.217	16 (3.7)	2 (2.6)	45 (20.4)	< 0.001
Level 2	450 (58.7)	170 (49.4)	280 (66.2)	< 0.001	225 (52.2)	51 (66.2)	153 (69.2)	< 0.001
Level 3	253 (33.0)	150 (43.6)	103 (24.4)	< 0.001	190 (44.1)	24 (31.2)	23 (10.4)	< 0.001
Anxiety/Depression, *n* (%)								
Level 1	383 (49.7)	147 (42.5)	236 (55.7)	< 0.001	187 (43.3)	31 (39.7)	151 (68.3)	< 0.001
Level 2	327 (42.5)	161 (46.5)	166 (39.2)	0.039	201 (46.5)	40 (51.3)	65 (29.4)	< 0.001
Level 3	60 (7.8)	38 (11.0)	22 (5.2)	0.003	44 (10.2)	7 (9.0)	5 (2.3)	0.001

*Level 1 = not impacted; Level 2 = moderately impacted; Level 3 = severely impacted

### Secondary outcomes: Absenteeism and presenteeism

Overall, 43.3% (*n* = 334) of patients reported at least one full day missed (mean = 9 full days) and 29.4% (*n* = 227) reported at least one partial day missed (mean = 6 partial days) (Additional file 1: Appendix 1); 433 patients (56.1%) reported at least one full or partial day of lost work (Table 3). Among the 695 patients that reported their effectiveness at work, only 19.1% were 100% effective during their HZ episode. There were 375 respondents (54.0%) who met the presenteeism definition (≤ 70%). Patients who considered work loss were more likely to have missed full (76.4% vs 26.0%, *p* < 0.001) or partial (70.9% vs. 35.2%, *p* < 0.001) days: 38.3% used sick days, 11.8% vacation, and 2.2% disability. Most common reasons for absence were the following: too uncomfortable (39.3%), too much pain (38.3%), and healthcare visits (34.6%). Mean EQ-5D scores for those who reported no work loss [0.79 (SD 0.18)] were higher than those with absenteeism [0.56 (SD 0.27)] or presenteeism [0.62 (SD 0.25)] (*p* < 0.001) (Table 3). While significant differences were observed across all EQ-5D domains, the largest impact of absenteeism/presenteeism was observed for ‘usual activities’ and ‘pain/discomfort’.

Among the entire study cohort, the odds of absenteeism/presenteeism increased significantly with increasing levels of HZ severity (Table 4). Higher odds of absenteeism/presenteeism were associated with pain located on the face/scalp/neck/eye/ear (OR = 1.90, 95% CI 1.06–3.40). The odds of absenteeism/presenteeism were higher among those with pain lasting 12+ months (OR = 2.91, 95% CI 1.14–7.42) or those who were currently employed (OR = 1.66, 95% CI 1.06–2.61), and were lower among males (OR = 0.58, 95% CI 0.40–0.84). Among the employed subset of patients, results were similar to those observed in the entire cohort with respect to the effect of HZ severity on absenteeism/presenteeism and male gender. However, among the employed subset, there was a marginal effect of pain located on the face/scalp/neck/eye/ear.

**Table 4:** Odds of absenteeism/presenteeism vs. no work loss (entire cohort, employed subset)

Independent Variables	Absent/Present vs. No Work loss, Entire Cohort (*N* = 722)				Absent/Present vs. No Work loss, Employed Subset (*N* = 584)			
	odds ratio	lower 95% CI	upper 95% CI	*p*-value	odds ratio	lower 95% CI	upper 95% CI	*p*-value
Herpes Zoster Severity								
Very mild	ref.	–	–	–	ref.	–	–	–
Mild	2.396	1.401	4.098	0.001	2.872	1.562	5.281	< 0.001
Moderate	5.707	3.312	9.834	< 0.001	6.683	3.593	12.430	< 0.001
Severe	7.279	3.880	13.658	< 0.001	9.293	4.478	19.284	< 0.001
Extremely severe	8.988	3.562	22.680	< 0.001	21.341	5.469	83.271	< 0.001
Location of pain^a^								
Shoulder/Upper Arm/Elbow/Forearm/Hand	0.831	0.488	1.415	0.496	0.858	0.450	1.636	0.643
Thigh/Knee/Shin/Calf/Ankle/Foot	0.809	0.397	1.652	0.561	0.811	0.365	1.801	0.607
Face/Scalp/Neck/Eye/Ear	1.895	1.056	3.399	0.032	1.955	0.997	3.832	0.051
Chest/Abdomen/Upper Back/Lower Back	1.474	0.852	2.550	0.166	1.553	0.827	2.916	0.171
Groin area/Buttocks	1.621	0.766	3.429	0.206	1.192	0.522	2.722	0.676
Length of time pain lasted categories								
Less than 1 month	ref.	–	–	–	ref.	–	–	–
1 to less than 3 months	1.490	0.952	2.334	0.081	1.416	0.850	2.357	0.181
3 months to less than 6 months	1.493	0.705	3.162	0.295	2.259	0.854	5.970	0.100
6 months to less than 12 months	1.412	0.521	3.827	0.498	1.306	0.406	4.198	0.655
12+ months	2.907	1.140	7.415	0.025	0.803	7.854	0.114	0.803
Currently employed	1.664	1.062	2.609	0.026				
Age (claims-based)	0.973	0.930	1.017	0.223	0.973	0.923	1.025	0.301
Gender								
Male	0.582	0.404	0.840	0.004	0.443	0.291	0.673	< 0.001
Female	ref.	–	–	–	ref.	–	–	–

^a^Subjects can report more than one location

## Discussion

Fewer than half of survey respondents with HZ indicated they considered work/income loss when responding to EQ-5D items, and EQ-5D scores were significantly associated with consideration of income loss. Patients that *did consider* work/income loss were more significantly impacted by their HZ. They reported more severe HZ, pain lasting more than 12 months, location of HZ on face, neck or scalp, and higher pain levels. More than half of patients with HZ (56.1%) reported at least one full or partial day of lost work due to HZ during the most recent shingles episode. Patients with absenteeism during their HZ episode were almost 8 times more likely to consider work/income loss when completing the EQ-5D.

As noted, among our study population of patients with predominantly (65%) mild to moderate HZ, 45% did not incorporate consideration of work or income loss in their EQ-5D response. Published research of patient work/income loss consideration for health-state utility assessment, conducted almost exclusively ex-US, indicates mixed evidence, as a majority of previous studies which have examined this issue across disease states report that fewer than 50% of respondents considered impact of work/income loss [19–26], while 2 studies found that most study participants considered work/income loss [27, 28]. Previous study results are mixed as to whether lost income affects utility scores, with income consideration more often associated with lower utilities for severely impaired health states and not mild [15, 19, 20, 28, 36]. In the US, Meltzer and colleagues requested 402 subjects to value back pain and 429 subjects to value blindness using the TTO method, and concluded that the economic costs of illness are not likely to be reflected in quality of life questions and should be counted separately [37]. However, since the study was only published in abstract form, with limited information provided regarding the survey method, the scale of TTO values, and background characteristics of the study sample [15], the generalizability and validity of the study’s findings to the association between work/income loss and health-state utilities in the US may be questionable.

A significant difference in mean EQ-5D index scores between the respondents in our study who *did consider* and *did not consider* work and/or income loss (0.56 vs. 0.69, respectively; *p* < 0.001) was observed, and respondents who considered work/income loss also reported greater HZ severity and duration of pain. These findings reflect the impact of HZ on health status that has previously been reported in the literature. Generally, studies which have employed the EQ-5D among patients with HZ have found an inverse association between health-related quality of life and levels of reported pain [38–40], with the poorest quality of life observed among patients with PHN [38, 39]. Across 6 European countries, van Seventer et al. examined the relationship between severity of PHN and health-related utility (EQ-5D), and found decreasing mean EQ-5D index scores among patients with mild, moderate and severe pain, respectively (mean EQ-5D: mild = 0.72, moderate = 0.63, and severe = 0.27) [40].

In the current study, 56.1% of patients with HZ reported at least one full or partial day of lost work due to HZ. Patients with absenteeism during their HZ episode were about 7.9 times more likely to consider work/income loss when completing the EQ-5D than were patients with only presenteeism or no work loss. Consistent with these findings, Singhal and colleagues, in a telephone survey of US patients identified in an administrative claims database, found that about half (51%) reported missing work due to HZ, and about an equal percentage reported little or much worse productivity than usual due to HZ while at work [4]. Drolet et al. [3] studied patients aged 50 years and older with HZ in Canada, and found that the majority (64%) missed work and that 76% reported decreased effectiveness at work due to HZ and/or PHN Studies by Weinke [41] (Germany) and Lukas [42] (Europe) also found that work loss is an important issue for HZ patients, as approximately 50% of HZ patients who were employed at the time each study was conducted reported absences due to HZ/PHN. Since we were unable to identify any published studies that directly addressed the question of the impact of work loss on quality of life in HZ, it is unknown whether HZ patients experiencing work loss rate themselves in worse health (i.e., map themselves into more severe EQ-5D health states) compared to other respondents who did not miss work, but are similar clinically.

It is important to consider our study’s results in the context of several limitations. Generalizability of the results of this study may be limited, as our patient population was identified from one commercial managed care health plan, and thus may not be applicable to patients with other health care coverage. The health plan used for our analysis included a wide geographic distribution of patients across the United States, and therefore, should provide the capability for generalization to commercially insured managed care populations on a national level. However, 91% of patients in the current study were white, and we could not ascertain whether this is representative of health plan enrollment or if it represented response bias; regardless, our patient population is not representative of the racial composition of the US as a whole. Administrative claims are collected for payment rather than research purposes, and thus have some limitations related to the accuracy of capture of patient medical and pharmacy use history, and may also be subject to coding errors. Finally, patient survey data may be subject to recall bias, and it is also possible that patients with more severe HZ may be more likely to respond to the mailed survey.

## Conclusions

HZ has considerable impact on the work and productivity of adults aged 50–64 years old. Consideration of lost time in paid work is associated with lower health status scores when patients complete a standardized quality of life questionnaire (e.g., EQ-5D). However, many patients with HZ do not spontaneously consider work/income loss when completing a standardized quality of life questionnaire. Studies that use health-state utilities in HZ, including those based on standardized scales such as EQ-5D, may not fully reflect the societal costs and consequences of work loss. Our study’s findings suggest that it may be appropriate to account for this effect separately in cost calculations.

## Additional file

Additional file 1: **Appendix.** Work absenteeism and presenteeism by considered work loss. Table describing absenteeism and presenteeism, productivity, and work loss. (DOCX 18 kb)

## Abbreviations

B: Billion
CI: Confidence interval
EQ-5D: EuroQol Five Dimensions Questionnaire
HIPAA: Health Insurance Portability and Accountability Act
HZ: Herpes zoster
ICD-9-CM: International Classification of Diseases, Ninth Revision, Clinical Modification
OR: Odds ratio
PHN: Post-herpetic neuralgia
SD: Standard deviation
