# Supplementary material for: A cross-sectional survey of work and income loss consideration among patients with herpes zoster when completing a quality of life questionnaire
Source: BMC Health Serv Res. 2018 Aug 25;18:662. doi: 10.1186/s12913-018-3451-9 (PMC6109314; doi:10.1186/s12913-018-3451-9)
Supplement: Supplementary file 1 — Appendix. Work absenteeism and presenteeism by considered work loss. Table describing absenteeism and presenteeism, productivity, and work loss. (DOCX 18 kb) [file 12913_2018_3451_MOESM1_ESM.docx]

Appendix 1. Work absenteeism and presenteeism by considered work loss

|  | **Total (N=772)** | | | **Considered = YES (N=347)** | | | **Considered = NO (N=425)** | | | **p-value** |
| --- | --- | --- | --- | --- | --- | --- | --- | --- | --- | --- |
|  | n | mean | SD | n | mean | SD | n | mean | SD |  |
| Missed work |  |  |  |  |  |  |  |  |  |  |
| Full days missed, of individuals that missed at least 1 full day | 334 | 9.39 | 17.06 | 240 | 9.12 | 13.71 | 94 | 10.09 | 23.63 | 0.709 |
| Partial days missed, of individuals that missed at least 1 partial day | 227 | 6.11 | 11.73 | 134 | 6.56 | 11.76 | 93 | 5.47 | 11.72 | 0.494 |
| Effectiveness at work (%) | valid N | n | % | valid N | n | % | valid N | n | % | p-value |
| 0% | 695 | 66 | 9.50 | 332 | 34 | 10.24 | 363 | 32 | 8.82 | 0.522 |
| 10% | 695 | 12 | 1.73 | 332 | 7 | 2.11 | 363 | 5 | 1.38 | 0.460 |
| 20% | 695 | 22 | 3.17 | 332 | 13 | 3.92 | 363 | 9 | 2.48 | 0.280 |
| 30% | 695 | 35 | 5.04 | 332 | 27 | 8.13 | 363 | 8 | 2.20 | <0.001 |
| 40% | 695 | 29 | 4.17 | 332 | 18 | 5.42 | 363 | 11 | 3.03 | 0.115 |
| 50% | 695 | 62 | 8.92 | 332 | 37 | 11.14 | 363 | 25 | 6.89 | 0.049 |
| 60% | 695 | 61 | 8.78 | 332 | 44 | 13.25 | 363 | 17 | 4.68 | <0.001 |
| 70% | 695 | 88 | 12.66 | 332 | 43 | 12.95 | 363 | 45 | 12.40 | 0.826 |
| 80% | 695 | 97 | 13.96 | 332 | 47 | 14.16 | 363 | 50 | 13.77 | 0.884 |
| 90% | 695 | 90 | 12.95 | 332 | 27 | 8.13 | 363 | 63 | 17.36 | <0.001 |
| 100% | 695 | 133 | 19.14 | 332 | 35 | 10.54 | 363 | 98 | 27.00 | <0.001 |
| Reason for absence |  |  |  |  |  |  |  |  |  |  |
| Healthcare visits | 682 | 236 | 34.60 | 338 | 132 | 39.05 | 344 | 104 | 30.23 | 0.015 |
| Unable to concentrate | 682 | 144 | 21.11 | 338 | 108 | 31.95 | 344 | 36 | 10.47 | <0.001 |
| Too much pain | 682 | 261 | 38.27 | 338 | 193 | 57.10 | 344 | 68 | 19.77 | <0.001 |
| Visible rash | 682 | 115 | 16.86 | 338 | 88 | 26.04 | 344 | 27 | 7.85 | <0.001 |
| Too uncomfortable | 682 | 268 | 39.30 | 338 | 190 | 56.21 | 344 | 78 | 22.67 | <0.001 |
| Side effects of shingles medication | 682 | 65 | 9.53 | 338 | 47 | 13.91 | 344 | 18 | 5.23 | <0.001 |
| NA (no absences) | 682 | 225 | 32.99 | 338 | 42 | 12.43 | 344 | 183 | 53.20 | <0.001 |
| Use of time off |  |  |  |  |  |  |  |  |  |  |
| Sick days | 772 | 296 | 38.34 | 347 | 211 | 60.81 | 425 | 85 | 20.00 | <0.001 |
| Disability | 772 | 17 | 2.20 | 347 | 11 | 3.17 | 425 | 6 | 1.41 | 0.098 |
| Vacation | 772 | 91 | 11.79 | 347 | 65 | 18.73 | 425 | 26 | 6.12 | <0.001 |
| No time off used or missing | 772 | 440 | 56.99 | 347 | 113 | 32.56 | 425 | 327 | 76.94 | <0.001 |
